# Mechanochemistry: An Efficient Way to Recycle Thermoset Polyurethanes

**DOI:** 10.3390/polym14163277

**Published:** 2022-08-11

**Authors:** Ping He, Hao Lu, Haoda Ruan, Congyang Wang, Qiang Zhang, Zezhong Huang, Jing Liu

**Affiliations:** College of Mechanical and Electrical Engineering, Anhui Jianzhu University, Hefei 230601, China

**Keywords:** mechanochemical method, recycled polyurethane foam, orthogonal test, tensile strength, thermal conductivity

## Abstract

A recycling process of waste thermosetting polyurethane plastics was proposed based on the mechanochemical method, aiming at the three-dimensional network cross-linking structure of thermosetting polyurethane. Orthogonal experimental design was adopted to select three factors of crushing speed, crushing time, and feed amount to determine the best crushing parameters. Then, the waste polyurethane insulation boards were crushed and degraded by the mechanism of regenerative forming with the adjustable speed test machine. Accordingly, the recycled powder was obtained. Finally, nine kinds of polyurethane recycled composite plates were prepared by hot pressing process. The degradation effect of thermosetting polyurethane was analyzed by Fourier transform infrared spectroscopy, scanning electron microscope, and X-ray diffraction. Moreover, the mechanical properties and thermal insulation properties of recycled composite plates were tested and analyzed. The results show that the network cross-linking molecular structure of waste thermosetting polyurethane plastics is destroyed by the effect of mechanochemical action, and methyl and aldehyde groups are decomposed. Therefore, a recycled powder with strong reactivity and plasticity is generated, which improves the activity regeneration ability. After adding thermoplastic resin, the mechanical properties and formability of recycled composite plates are enhanced, with maximum tensile strength up to 9.913 MPa. Correspondingly, the thermal insulation performance of plates is reduced. However, the minimum thermal conductivity can also reach 0.056 W/m·K. This study provides an effective method for the recycling of thermosetting polyurethane plastics.

## 1. Introduction

Due to its good stability and corrosion resistance, thermosetting polyurethane has become a key application in construction, automobiles, coatings, medical equipment, clothing, aviation, and other aspects [1]. However, with the production and consumption of polyurethane, a huge amount of polyurethane waste is generated. How to deal with the waste polyurethane has become an important practical problem for energy saving, environmental protection, and sustainable economic development [2]. Furthermore, thermosetting polyurethane forms a cross-networking structure after heating and curing, making it insoluble and unable to melt and regenerate like thermoplastics. If buried directly, it is easy to produce secondary pollution while wasting resources [3]. Therefore, scholars all over the world are studying how to properly recycle thermosetting polyurethane.

There are three main recovery methods of thermosetting polyurethane: the physical recovery method, the chemical recovery method, and the thermal recovery method [4,5,6]. At present, all of them have been applied in industrial production, which plays an important role in improving the recovery rate of thermosetting polyurethane foam and improving environmental pollution. However, there are still some problems in practical application: the physical recovery method [7] has the advantages of simple operation and low cost, but the powder activity is low. This leads to a limited range of recycled products and a low economic value obtained after recovery [8].

Chemical recovery follows the principle of degradation [9], so it has high requirements for reaction equipment and conditions. At present, most decomposition methods are still in the laboratory stage [10]. Although glycolysis [11] has been officially put into production, it is difficult to expand the industrial scale of glycolysis due to the high cost of separation and purification and the high toxicity of aromatic amines, a byproduct of glycolysis.

The thermal energy recovery method [12], also known as the incineration method, takes polyurethane waste as fuel and burns it to recover energy, which is also the main treatment method for domestic waste at present. However, it is worth mentioning that polyurethane, as a common thermal insulation material, is often added with flame retardants to improve fire performance [13]. This causes polyurethane waste to fail to burn adequately. Incomplete combustion of polyurethane will produce toxic gases (such as CO, NOx, etc.), causing serious environmental pollution.

To sum up, there is an urgent need for high-value and low-cost thermosetting polyurethane recycling methods in today’s society. The mechanochemical method can meet this need.

Mechanochemistry refers to the phenomenon of chemical reactions of substances caused by the input of mechanical energy (such as crushing in a ball mill) [14]. It has been studied more and more thoroughly, partly because it can promote reactions between solids quickly and easily, with little or no solvent added [15]. The earliest recorded mechanochemical experiments were performed in the 4th century BC by crushing cinnabar with acetic acid in a copper vessel to obtain elemental mercury. Since then, the mechanochemical reaction has also been used in gold mining and metallurgy as an auxiliary means of chemical synthesis. It was not until the early 20th century that Wilhelm Ostwald formally named it mechanochemistry and classified it as one of the four subdisciplines of chemistry. In the decades that followed, however, mechanochemistry was only a substitute for solvent synthesis, and was only applied to insoluble inorganic materials. It was after the 1980s that mechanochemistry was significantly developed after Patil, Toda et al. [16] applied mechanochemistry to eutectic preparation. At present, there is extensive research on inorganic materials, co-crystallization, organic synthesis, supramolecular, and so on.

The discovery that the mechanochemical method can directly break polymer molecular bonds [17] provides an important idea for the recycling of thermosetting plastics. Under the action of long-time mechanical force (such as cutting, crushing, impact, etc.), mechanical energy and heat energy accumulate continuously, which causes the main chain fracture of the polymer at the center, the molecular structure is destroyed, and the polymer activity is enhanced. In view of this characteristic, Hu et al. [18] studied the recovery mechanism of thermosetting phenolic resin by the mechanochemical method and optimized the recovery process parameters. Under the optimal process parameters (the speed is 2820 r/min, the time is 80 min, the feed size is 0.43 mm, the feed volume is 60 g), the tensile strength and bending strength of the recycled material could reach 8.13 MPa and 17.76 MPa.

In this paper, the recycling method of thermosetting polyurethane based on the mechanochemical reaction was studied using a self-made pulverizer. By changing the crushing speed, crushing time, and feed amount, polyurethane crushing experiments were carried out to determine the best crushing parameters. Crushing and regeneration experiments were conducted on rigid polyurethane insulation boards. The experimental process is accompanied by a variety of mechanical forces such as shear, extrusion, crushing, and friction. Due to the accumulation of mechanical energy, the internal stress is not uniformly distributed, or the impact energy is concentrated on individual chain segments, which generates critical stress to break the chemical bonds. At the same time, the thermal energy generated by the mechanical force prompts a certain degree of fracture of the chemical bonds in the molecular structure with weak bonding energy. It destroys the cross-linked structure of polyurethane insulation board macromolecules, enhances the activity of functional groups, and reduces the cross-linked density. The obtained powder is more active, which makes the polyurethane powder recover certain plasticity and regain the ability of processing and forming. Blended recycled thermoplastic polypropylene powder, no other chemical additives were added considering the need for green recycling and cost control. Finally, polyurethane recycled composite panels with different particle sizes and mass ratios were prepared by heat press molding process. The mechanical properties, forming ability, and thermal insulation properties of the recycled composite panels were tested to obtain the optimal performance parameters of the recycled composite panels. The research results provided a basis for the recovery of waste thermosetting polyurethane.

## 2. Materials and Methods

### 2.1. Experimental Materials

**Figure 1:**
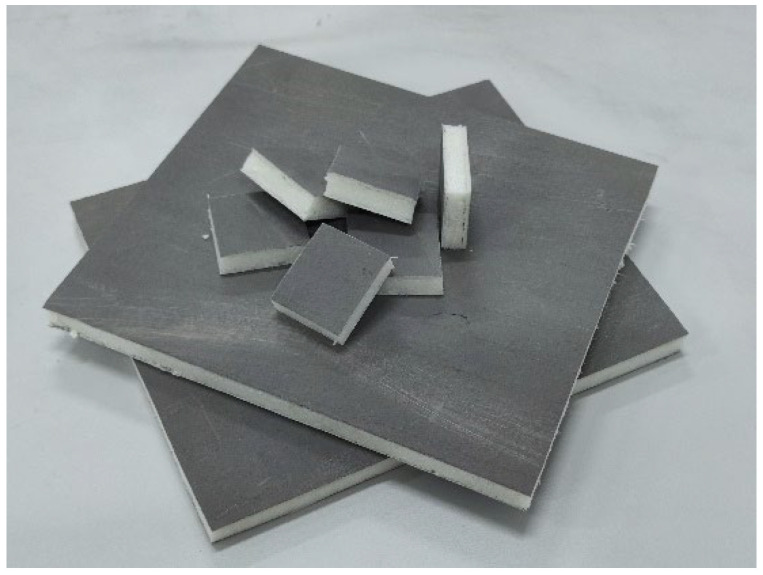
Waste rigid polyurethane insulation board.

The waste polyurethane foam used in this study is rigid polyurethane insulation board (Langfang, Hebei, China). As shown in Figure 1, the outer side of this board is a fireproof layer composed of non-woven fabric and inorganic paste, and the middle is rigid polyurethane foam. It is one of the most commonly used thermal insulation materials in the construction industry. The matrix material (recycled polypropylene) is from ZhongLian Plastic Corp. (Dongguan, Guangdong, China).

### 2.2. Experiment Process

#### 2.2.1. Comminution Process

Firstly, the attachment on the surface of waste polyurethane foam was removed, cleaned, dried, and manually divided into 5 cm^2^ pieces, then the particles were roughly crushed to less than 5 mm. To fit the actual recovery conditions, the fireproof layer was retained. It can enhance the strength of the recycled broad and improve the economic benefit. The particles were finally crushed into powder with a self-made crusher. The speed of crusher was set to 3000–5000 r/min, the crushing time was 10–30 min, and the feed quantity was 50–90 g. Based on orthogonal test design table L9 (3^4^), a total of 9 groups of tests were conducted, as shown in Table 1, with each row representing one test. D column is a blank column, also known as an error column, used to calculate test error.

#### 2.2.2. Evaluation System of Crushing Effect

The purpose of choosing the mechanochemical method to recover thermosetting plastics is to obtain a highly active plastic powder. According to the principle of mechanochemical reaction, long-term mechanochemical action can break polyurethane molecular bonds to enhance powder activity, and its macroscopic performance is the increase in specific surface area of the powder. The specific surface area could not be measured directly, so the powder ratio of 200 mesh was used as one of the evaluation indexes of the comminution effect of thermosetting polyurethane. At the same time, it is not desirable to pursue powder activity blindly. To meet the needs of large-scale industrial applications, energy consumption in actual production should be considered. Because the ratio of 200 mesh powder is the most promising index, the energy saving rate is used as another index to evaluate the comminution effect.

The evaluation indexes of crushing effect of thermosetting polyurethane are defined as follows:

(1) $\alpha =m_{2}/m_{1}$, where $m_{2}$ is less than 200 mesh polyurethane powder mass, $m_{1}$ is the total mass.

(2) $\beta =\left(W_{1}-W_{2}\right)/W_{1}$, where $W_{1}$ is the energy consumed in 30 min at rated power, and $W_{2}$ is the energy consumed in a single experiment measured by an energy meter.

Evaluation function of comminution effect of thermosetting polyurethane was established by linear weighting method:

(1) $$Y=\overset{2}{\underset{i=1}{\sum }}k_{i}f_{i}=k_{1}\alpha +k_{2}\beta$$

where *k* is the weight.

The multi-decision-maker scoring method is adopted to assign the basic weights of the above two evaluation indicators based on the judgment of importance. The sum of the weights is 1 and the minimum is 0. The number of weights is shown in Table 2.

The mean of the basic weight is not the final weight number, and the decision maker’s score is based on the importance of the evaluation index rather than the specific value of the evaluation index. When the value range of an evaluation index is several times that of other evaluation indexes, the weight should be modified based on its numerical proportion.

#### 2.2.3. Heat Press Process

Before the molding test, a layer of PET film was laid on the bottom of the mold to prevent the melt from bonding with the mold. The thermosetting polyurethane powder and recycled PP powder were evenly mixed in a mixer and laid in the mold. Due to the large packing density of polyurethane powder and polypropylene powder, in order to ensure the transfer of pressure, pressing the die was pressed several times after compaction powder filling, to ensure that the height of the powder in the concave die could reach about 9 mm. Then, a layer of PET film was put on the powder, and finally, the punch was covered.

Polyurethane powders of 40, 120, and 200 mesh sizes were taken and molded by three adding ratios of 50%, 65%, and 80%, respectively. The mold was preheated at 175 °C for 10 min before each experiment. Then, hot pressing at 195 °C for 30 min. After the hot pressing, there was the first pressure relief exhaust, and then pressure insulation for 10 min. Finally, the mold was removed and cooled to room temperature before demolding. The size of the final the board was about 150 × 150 × 5 mm^3^.

### 2.3. Laboratory Instruments

#### 2.3.1. Mill

The main components include motor, fan, dust removal bin, control panel, hopper, collecting bin, and crushing parts (multi-blade tooth rotor, crushing disc, crushing bin). It can provide shear, impact, crushing, and other mechanical force loadings, and can achieve 600–5000 r/min at infinitely variable speed. After the mill is started, the rotor rotates at high speed, and the material is first subjected to the strong shear force of the rotor cutter teeth, spreads around at high speed, and is subjected to the impact force of the feedback of the crushing bin and the friction of the crushing disc. Under the accumulation of a large amount of mechanical energy and heat energy, thermosetting plastics are completed with mechanochemical degradation.

#### 2.3.2. Plate Vulcanizing Machine

Polyurethane powder was remolded with a flat vulcanizing machine XLB350X (Qicai Hydraulic Machinery Corp., Shanghai, China). During the molding process, the temperature was set at 185–205 °C for 30–50 min, and the proportion of polyurethane powder was 50–80%.

#### 2.3.3. Fourier Infrared Spectroscopy Analysis

The functional group structure and chemical bond changes of polyurethane powder were measured by Fourier transform infrared spectrometer Nicolet iS50 (Thermo Fisher Scientific Inc., Waltham, MA, USA). Powder of 3 mesh (40, 120, 200) was added into potassium bromide to make a tablet and scanned 32 times for determination.

#### 2.3.4. X-ray Diffraction Analysis

Phase and microstructure changes of polyurethane powders were analyzed by X-ray diffractometer D8 Advance (Bruker Cor., Karlsruhe, Germany) at a scanning Angle of 5–90° and a scanning speed of 1°/min on a copper target.

#### 2.3.5. Scanning Electron Microscope (SEM)

The microstructure of polyurethane powders was observed using a scanning electron microscope EVO-18 (Carl Zeiss AG, Oberkochen, Baden-Wurttemberg, Germany). Before observation, gold plating was carried out under vacuum, and the acceleration voltage was selected as 20 kV.

#### 2.3.6. Tensile Strength

Materials testing System AGS-X (Shimadzu Corp, Kyoto, Japan) was used to test the tensile strength of recycled boards. The regeneration plate was cut into a dumbbell shape and the test speed was 1 mm/min. The experiment was repeated three times for each group and the average value was taken.

#### 2.3.7. Thermal Conductivity

Heat flow meter apparatus DRPL-III (XiangYi, Instrument Co., Ltd., Xiangtan, Hunan, China) was used to detect the thermal conductivity of nine boards. The cold surface was set to 25 °C, the hot surface to 40 °C, and the pressure to 80 N.

## 3. Results and Discussion

### 3.1. Results of the Particle Size Distribution of the Polyurethane Powder

**Figure 2:**
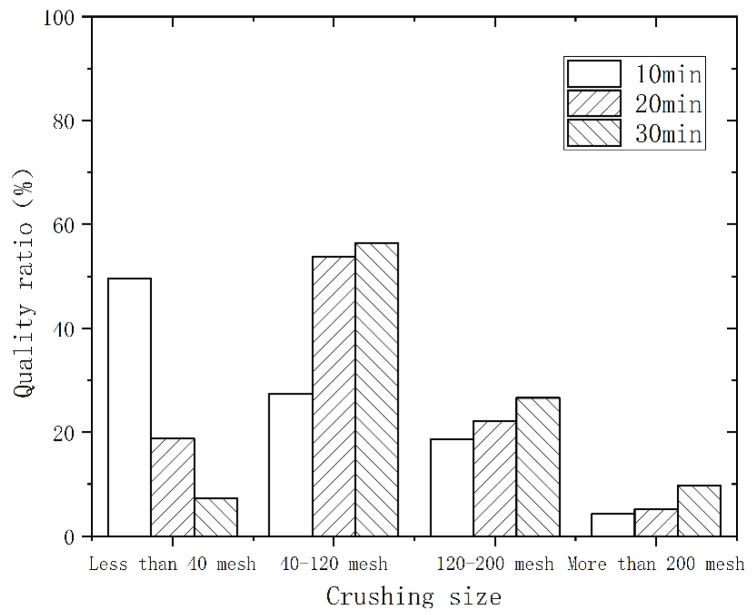
Particle size distribution for different times at 3000 r/min.

**Figure 3:**
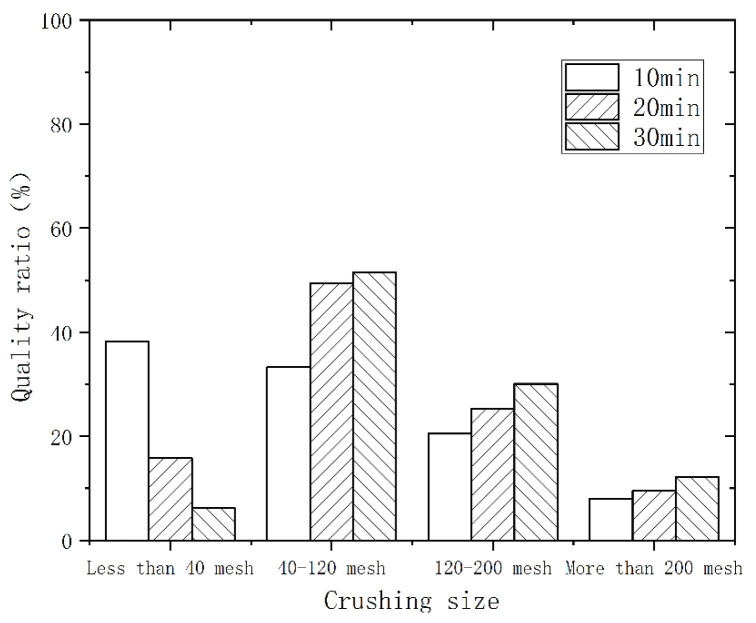
Particle size distribution for different times at 4000 r/min.

**Figure 4:**
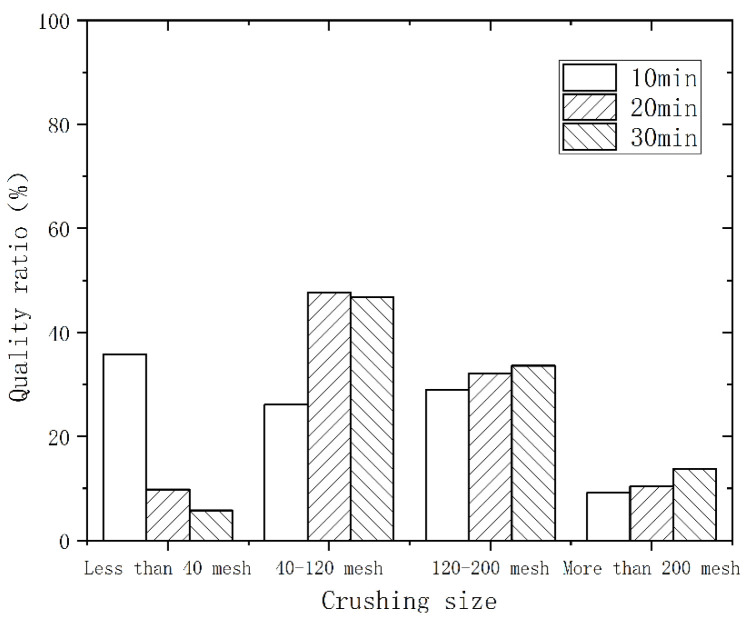
Particle size distribution for different times at 5000 r/min.

As the speed rose and the crushing time increased, the polyurethane powder evolved from a large flake structure to a round and small flake structure. Figure 2 shows the particle size distribution at different times with a speed of 3000 r/min. The vertical coordinate is the percentage of the total mass of the material in each particle size interval. With the extension of the crushing time, the material was further refined. In the range above 200 mesh, the mass ratio increased from 4.3% to 9.7%. Figure 3 and Figure 4 show the particle size distribution at speeds of 4000 r/min and 5000 r/min, respectively. At a crushing time of 30 min, the speed increased from 3000 r/min to 5000 r/min, and the mass ratio decreased from 56.4% to 46.8% in the range of 40–120 mesh, while the mass ratio increased from 9.7% to 13.8% in the range of more than 200 mesh. Therefore, extending the time and increasing the speed can enhance the crushing and regeneration effect, and the crushed material is finer.

### 3.2. Results of Comminution Experiment

The results of the comminution experiment are shown in Table 3. According to the sum of each evaluation index, the weight was modified.

Table 4 shows the analysis results of the effect range. Ki is the sum of the values of the crushing effect at level i under a factor, and ki is the average of the i-th level of the factor. According to the R-value, each factor is: B > A > C, indicating that the crushing time has the greatest influence on the crushing effect, followed by the speed of the crusher, and the feed quantity has a little influence. From the range analysis results, the optimal level combination of various factors for the crushing effect could be obtained: A3B3C3, which is the crusher with a speed of 5000 r/min, the crushing time is 30 min, and the feed amount is 90 g. The corresponding experimental results at this point are: 200 mesh powder is 19.02; energy saving rate is 18.11; crushing effect is 13.985.

According to the results in Table 3, analysis of variance (ANOVA) was conducted on the crushing effect, and the statistical results are listed in Table 5. SS is the sum of squares of variables; DOF represents degrees of freedom; MS is the mean square, that is, the ratio of SS to DOF; F and *p* are the values that determine whether a variable is significant. The higher the F value is, the lower the *p* value is, indicating that the variable is more significant. It can be seen from Table 5 that the results of ANOVA are consistent with the previous range analysis. Crushing time B has an extremely significant influence on the crushing effect, while the influence of feed amount on the crushing effect is very small and can be ignored.

In order to predict the trend of crushing effect, the horizontal coding of crushing speed A and crushing time B was selected as independent variables to fit the multiple quadratic regression model of crushing effect Y:

(2) $$Y=8.860+1.667\;A\;-\;1.464\;B\;-\;0.2285\;A^{2}+0.7235\;B^{2}\;-\;0.0302\;\mathrm{AB}$$

To test the fitting effect, ANOVA of the coefficients of the multiple quadratic regression model is shown in Table 6.

As can be seen from Table 6, the *p* value of the retrospective model is extremely small, so the multiple quadratic regression model is extremely significant. In addition, the determination coefficient R^2^ of the regression model was calculated by Formula (3):

(3) $$R^{2}=1-\frac{\mathrm{SSE}}{\mathrm{SST}}$$

where SSE is the sum of squares of errors and SST is the sum of squares of total deviations.

It can be seen from the above equation that the smaller the error is, the closer the value of R^2^ is to 1, proving that the regression model has a higher degree of data fitting. The value of R^2^ obtained by this calculation is 0.9979. Therefore, it is believed that this multiple quadratic regression model can accurately predict the crushing effect Y.

### 3.3. Powder Performance Analysis

#### 3.3.1. Fourier Infrared Spectroscopy Analysis

**Figure 5:**
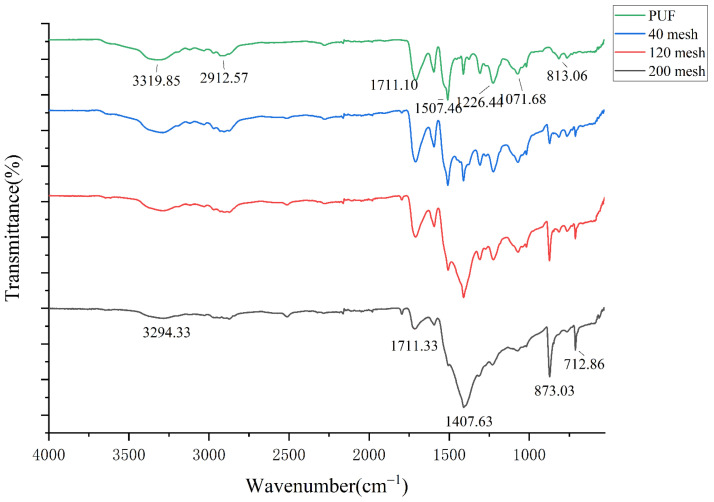
Fourier infrared spectra of polyurethane powders with different mesh numbers.

Fourier transform infrared spectroscopy (FTIR), by comparing the interference light and the control light of the sample, can obtain the transmittance of the sample under different wave numbers, to analyze the structure of the sample, with high sensitivity, good repeatability, fast scanning speed, and other advantages. The thermosetting polyurethane powder mentioned above was sieved into 40 mesh, 120 mesh, and 200 mesh, respectively. Fourier infrared spectrometer was used to analyze the powder of the three mesh numbers and the original rigid polyurethane foam, as shown in Figure 5.

Because polyurethane has more monomers, its infrared spectrum is more complex and there are more absorption peaks. Among them, the absorption peak of amino (-NH-) is at 3319.85 cm^−1^. With the increase of mesh number, the absorption peak here gradually weakened and widened, which is affected by the degradation of hydroxyl (-Oh-). The absorption peak of the amino (-NH-) decreased from 3311.18 cm^−1^ to 3294.33 cm^−1^ as the particle size of the powder increased from 40 to 200 mesh. As can be seen from the figure, the absorption peaks of methyl (-CH3-) and methylene (-CH2-) with wave number 2912.57 cm^−1^ and aldehyde group (C=O) with wave number 1711.10 cm^−1^ are the weakest with intensity at 200 mesh. At this time, the cross-linked structure of thermosetting polyurethane was damaged. In the infrared spectrum of 200 mesh powder, the wave number at 1407.63 cm^−1^ was the characteristic peak of isocyanate dimer, which was generated due to the destruction of the carbamate group. The enhancement of this absorption peak indicated that the mechanochemical effect could effectively destroy the crosslinking structure of polyurethane material and enhance the activity of powder.

#### 3.3.2. X-ray Diffraction Analysis

X-ray is the light radiation generated by the transition of electrons in the inner layer of atoms under the bombardment of high-speed moving electrons. By analyzing the diffraction pattern, analyzing the phase and microstructure changes of the material, the composition of the material and the structure information of the internal atoms or molecules can be obtained.

**Figure 6:**
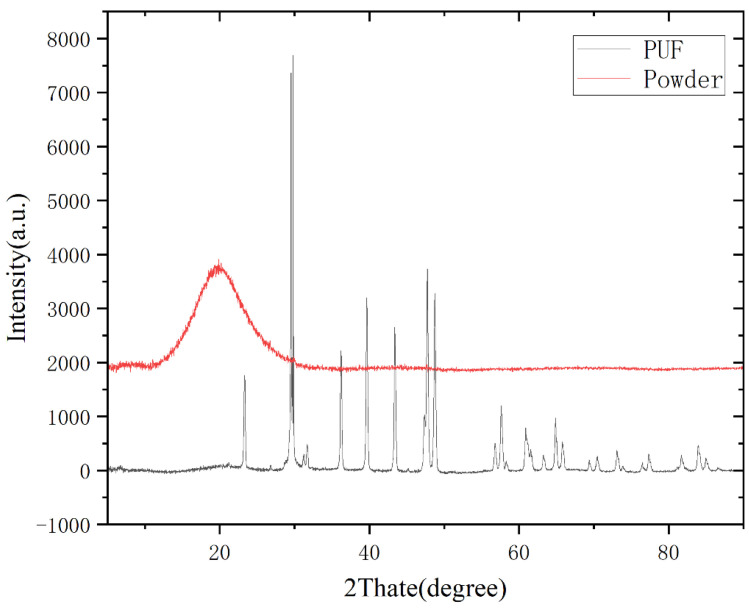
XRD curves before and after crushing.

Figure 6 shows the XRD patterns of waste thermosetting polyurethane before and after crushing. As the speed rises and the crushing time increases, the crushing particle size increases and several sharp diffraction peaks disappear. As the crushing proceeds, the particle size decreases and the fronts become wider, with a diffuse diffraction peak appears at 20°. It means that the structure of polyurethane is destroyed and loses its orderliness, and the vesicles fracture and break into an amorphous structure, which is the result of plastic deformation caused by mechanical force damage.

#### 3.3.3. SEM Analysis

Scanning electron microscopy was used to observe the microstructure of the sample. Considering the weak electrical conductivity of thermosetting polyurethane, a vacuum coating machine was used to gild the surface of polyurethane before the test. At the same time, the acceleration voltage 20 kV was selected, which can meet most material analysis requirements. Compared with the low acceleration voltage, the obtained image resolution is higher, and the depth of field effect is more obvious, which is conducive to the analysis of the internal morphology.

**Figure 7:**
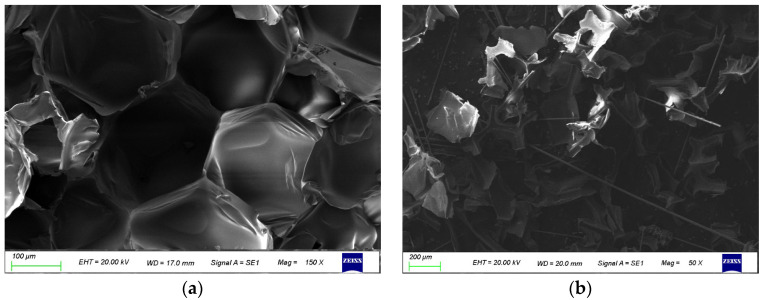
Microstructure of polyurethane before and after crushing, (**a**) before crushing, (**b**) after crushing.

Figure 7 shows the microstructure of polyurethane before and after crushing. As mentioned above, the test material selected in this paper is rigid polyurethane foam. From Figure 7a, it can be clearly seen that the cellular structure of polyurethane foam before crushing is the reason for its excellent insulation performance. Figure 7b shows the micromorphology of polyurethane after crushing. It can be seen that the polyurethane foam is mainly divided into two parts after crushing. The other is the position of the hole wall of the bubble hole, which is formed into a group after crushing, containing a part of air, which provides ideas for the regeneration of the polyurethane plate later.

In addition, it is worth noting that the picture of pulverized polyurethane powder contains a large number of fiber materials, which are from the fireproof layer material on the outer side of the rigid polyurethane insulation board mentioned above. To fit the actual recycling need, it was selected to not remove it. Retaining the fireproof layer can reduce the treatment cost, and the presence of fiber material can effectively improve the mechanical properties of the recycled board in the subsequent sheet forming.

**Figure 8:**
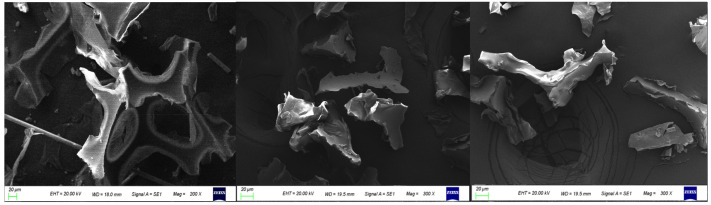
Microstructure of polyurethane powders with different mesh numbers: (**a**) 40 mesh, (**b**) 120 mesh, (**c**) 200 mesh.

In addition, the microstructure of powders of 40 mesh, 120 mesh, and 200 mesh were compared, as shown in Figure 8. It can be seen that the shape of 120 mesh and 200 mesh powder is relatively similar, while the 40 mesh powder retains a relatively complete bubble structure; so, in the plate forming, it can be considered to use this bubble structure to restore the thermal conductivity of polyurethane.

### 3.4. Performance Analysis of Board

#### 3.4.1. Tensile Strength Analysis

**Figure 9:**
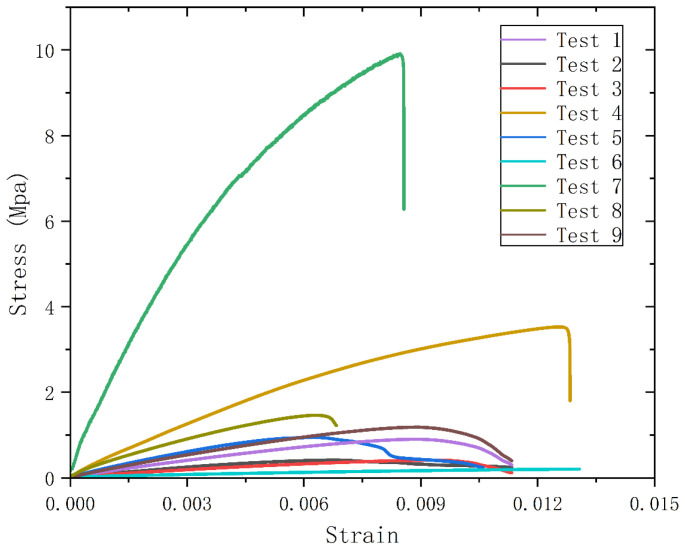
Stress versus strain curve.

Table 7 shows the experimental results of tensile strength, and the stress–strain curve is drawn as shown in Figure 9. From the results of the tensile strength test in Table 7, it can be seen that E and F have a significant effect on the tensile strength within a certain range. There is a tendency for the tensile strength of the specimen to decrease as F increases. When the addition ratio reached 50%, polyurethane became the main raw material for the specimen and the tensile strength reached 9.913 MPa. As shown in Figure 9, Test 7 remolded sheet has excellent mechanical properties, which is to be expected considering its process parameters (200 mesh, 50%). The 200 mesh polyurethane powder was crushed by mechanochemical action, and the mesh structure was broken. It had the highest degree of uncross-linking, and also had stronger surface activity, which could be better combined with polypropylene powder. In addition, the high mass ratio of polypropylene powder provides the basis for high tensile strength. Compared with the Test 4 remolded plate (120 mesh, 50%, 195 °C, 50 min), it can be seen that the polyurethane powder with high activity has great significance in the process of polyurethane remolding.

#### 3.4.2. Thermal Conductivity Analysis

To reduce the thermal contact resistance, the experimentalists polished and refined the sample surface. The initial surface temperatures of the cold and hot plates were 25 °C and 40 °C, respectively, and the test pressure was 80 N. As shown in Table 8, the thermal conductivity of nine panels was measured and the average value of each group in the test was taken three times. The calculation formula of thermal conductivity is as follows:

(4) $$\lambda =0.5\left(f_{1}e_{1}+f_{2}e_{2}\right)\frac{d}{\Delta T}\;$$

where: λ is the thermal conductivity coefficient, f is the calibration factor, e is the output voltage of the heat flow meter, and d is the average thickness of the sample.

Table 8 lists the test results of the thermal conductivity. It can be seen that the influence of the variation of E and F on the thermal conductivity shows an opposite effect. When F is 50%, the thermal conductivity increases from 0.071 W/m·K to 0.126 W/m·K as E increases from 40 mesh to 200 mesh. When E is 40 mesh, the thermal conductivity decreases from 0.071 W/m·K to 0.056 W/m·K as F increases from 50% to 80%. At present, the thermal conductivity of insulation materials in the industry standard is less than 0.12 W/m·K, and the specimens meet the requirements except for the Test 7 plate. The thermal conductivity of the Test 3 plate is as low as 0.056 W/m·K, which is close to the standard of 0.05 W/m·K for efficient insulation materials. According to its parameters (40 mesh, 80%, 205 °C, 50 min), 40 mesh polyurethane powder provides strong thermal insulation ability for the recycled sheet by retaining the polyurethane foam structure. Compared with the Test 3 plate and the Test 7 plate, it is found that the thermal conductivity of the remolded plate is positively correlated with the tensile strength.

## 4. Conclusions

The following are the conclusions from this study:

The mechanochemical effect of polyurethane during long-time comminution was studied. After the accumulation of mechanical energy and heat energy, the crosslinking structure of polyurethane is damaged, and polyurethane gradually degrades to produce a large number of isocyanate dimers.

Taking the speed, crushing time, and feed amount of the crusher as the influencing factors, the ratio of 200 mesh powder and the energy retention rate as the index, the change of the crushing effect was studied, and the optimal process parameters were given as 5000 RPM/min, 30 min, 90 g.

Polyurethane powder and polypropylene were mixed and pressed into composite sheets with different mesh and proportions. The maximum tensile strength can reach 9.913 MPa, and the minimum thermal conductivity is 0.056 W/m·K, which has good performance and a strong application value.

## Figures and Tables

**Table 1:** Orthogonal experiment table L9 (3^4^).

Test No.	A (Speed)	B (Time)	C (Quantity)	D
1	3000	10	50	1
2	3000	20	70	2
3	3000	30	90	3
4	4000	10	70	3
5	4000	20	90	1
6	4000	30	50	2
7	5000	10	90	2
8	5000	20	50	3
9	5000	30	70	1

**Table 2:** Two-hundred mesh powder ratio index weight.

Decision Makers	200 Mesh Powder Ratio	Energy Saving Rate
1	0.65	0.35
2	0.7	0.3
3	0.6	0.4
4	0.75	0.25
5	0.7	0.3
average	0.68	0.32

**Table 3:** Results of the comminution experiment.

Test No.	A	B	C	200 Mesh Powder	Energy Saving Rate	Crushing Effect
1	1	1	1	6.20	82.41	9.572
2	1	2	2	8.34	68.43	10.119
3	1	3	3	13.24	51.75	12.367
4	2	1	2	8.00	76.59	10.418
5	2	2	3	11.30	54.69	11.239
6	2	3	1	15.82	37.03	13.164
7	3	1	3	9.31	71.69	10.990
8	3	2	1	12.56	46.43	11.559
9	3	3	2	18.14	20.44	13.664

**Table 4:** Range analysis of crushing effect.

	A	B	C
K1	32.059	30.982	34.296
K2	34.822	32.917	34.201
K3	36.213	39.195	34.596
k1	10.686	10.327	11.432
k2	11.607	10.972	11.400
k3	12.071	13.065	11.532
R	1.385	2.738	0.132

**Table 5:** ANOVA of crushing effect.

Variable	SS	DOF	MS	F	*p*	Significance
A	2.9818	2	1.49088	425.93	0.002	*
B	12.2946	2	6.14730	1756.21	<0.001	**
C	0.0284	2	0.01419	4.05	0.198	
Error	0.0070	2	0.00350			
Total	15.3118	8				

* Significant difference (*p* < 0.05); ** Extremely significant difference (*p* < 0.001).

**Table 6:** ANOVA of multiple quadratic regression model.

Variable	SS	DOF	MS	F	*p*	Significant
model	15.2800	5	3.05601	288.98	<0.001	**
A	0.3032	1	0.30315	28.67	0.013	*
B	0.2339	1	0.23392	22.12	0.018	*
A^2^	0.1044	1	0.10442	9.87	0.052	
B^2^	1.0469	1	1.04690	99.00	0.002	*
AB	0.0037	1	0.00366	0.35	0.598	
Error	0.0317	3	0.01058			
Total	15.3118	8				

* Significant difference (*p* < 0.05); ** Extremely significant difference (*p* < 0.001).

**Table 7:** Results of tensile strength test.

Test No.	E (Powder Mesh)	F (Powder Ratio)	Tensile Strength (MPa)
1	40	50%	0.903
2	40	65%	0.418
3	40	80%	0.414
4	120	50%	3.857
5	120	65%	0.965
6	120	80%	0.227
7	200	50%	9.913
8	200	65%	1.465
9	200	80%	1.187

**Table 8:** Results thermal conductivity test.

Test No.	E (Powder Mesh)	F (Powder Ratio)	Thermal Conductivity (W/m·K)
1	40	50%	0.071
2	40	65%	0.065
3	40	80%	0.056
4	120	50%	0.098
5	120	65%	0.073
6	120	80%	0.060
7	200	50%	0.126
8	200	65%	0.080
9	200	80%	0.061

## Data Availability

Data generated or analyzed during this study are included in this published article.
